# Evodiamine decreased the systemic exposure of pravastatin in non-alcoholic steatohepatitis rats due to the up-regulation of hepatic OATPs

**DOI:** 10.1080/13880209.2022.2036767

**Published:** 2022-02-16

**Authors:** Ruifeng Liang, Wenjing Ge, Bingjie Li, Weifeng Cui, Xiaofan Ma, Yuying Pan, Gengsheng Li

**Affiliations:** a Institute of Chinese Materia Medica, Henan Provincial Academy of Traditional Chinese Medicine, Zhengzhou, China; b School of Pharmacology, Henan University of Traditional Chinese Medicine, Zhengzhou, China

**Keywords:** Drug-drug interaction, pharmacokinetics, hepatic uptake, organic anion transporting polypeptide

## Abstract

**Context:**

Patients with non-alcoholic steatohepatitis (NASH) may have a simultaneous intake of pravastatin and evodiamine-containing herbs.

**Objective:**

The effect of evodiamine on the pharmacokinetics of pravastatin and its potential mechanisms were investigated in NASH rats.

**Materials and methods:**

The NASH model was conducted with feeding a methionine choline-deficient (MCD) diet for 8 weeks. Sprague-Dawley rats were randomised equally (*n* = 6) into NASH group, evodiamine group (10 mg/kg), pravastatin group (10 mg/kg), and evodiamine (10 mg/kg) + pravastatin (10 mg/kg) group. Normal control rats were fed a standard diet. Effects of evodiamine on the pharmacokinetics, distribution, and uptake of pravastatin were investigated.

**Results:**

Evodiamine decreased *C*_max_ (159.43 ± 26.63 vs. 125.61 ± 22.17 μg/L), AUC_0-t_ (18.17 ± 2.52 vs. 14.91 ± 2.03 mg/min/L) and AUC_0-∞_ (22.99 ± 2.62 vs. 19.50 ± 2.31 mg/min/L) of orally administered pravastatin in NASH rats, but had no significant effect in normal rats. Evodiamine enhanced the uptake (from 154.85 ± 23.17 to 198.48 ± 26.31 pmol/mg protein) and distribution (from 736.61 ± 108.07 to 911.89 ± 124.64 ng/g tissue) of pravastatin in NASH rat liver. The expression of Oatp1a1, Oatp1a4, and Oatp1b2 was up-regulated 1.48-, 1.38-, and 1.51-fold by evodiamine. Evodiamine decreased the levels of IL-1β, IL-6, and TNF-α by 27.82%, 24.76%, and 29.72% in NASH rats, respectively.

**Discussion and conclusions:**

Evodiamine decreased the systemic exposure of pravastatin by up-regulating the expression of OATPs. These results provide a reference for further validation of this interaction in humans.

## Introduction

Non-alcoholic steatohepatitis (NASH), the progressive stage of non-alcoholic fatty liver disease (NAFLD), is a prevalent chronic disease characterised by excessive fat accumulation and inflammation in the liver. As the most common chronic liver disease, NASH has been identified as a source of drug variability in patients and could have serious implications for the safety and efficacy of xenobiotics (Cobbina and Akhlaghi 2017). The elevation of pro-inflammatory cytokines, such as interleukin-1β (IL-1β), tumour necrosis factor-α (TNF-α), and interleukin-6 (IL-6), not only plays an important role in the disease state of NASH (Bocsan et al. 2017; Lambrecht and Tacke 2020) but may also regulate the expression of cytochrome P450 (CYP450) enzymes and transporters which alter the pharmacokinetics of various therapeutic drugs (Le Vee et al. 2009; Li et al. 2017).

Statins, the first-line pharmacotherapy for lowering low-density lipoprotein cholesterol, are commonly used by NAFLD patients including those suffering from NASH because approximately 69.16% of NAFLD patients have hyperlipidaemia (Younossi et al. 2016). Despite these drugs being incredibly effective and generally well-tolerated, it is estimated that as many as 40% of eligible patients have difficulty in adhering to statins treatment, with one of the major barriers being intolerance due to myopathy and/or hepatotoxicity (Bellosta and Corsini 2018). As the adverse reactions of statins are associated with plasma levels, understanding the mechanisms behind variable drug response is essential for predicting their safety and efficacy.

Pravastatin is a hydrophilic statin that is often used, which is not significantly metabolised by the cytochrome P450 (CYP450) system (Watanabe et al. 2009) and hepatobiliary excretion is considered to be the rate-limiting step of pravastatin clearance (Kalliokoski and Niemi 2009). It has been demonstrated that hepatic uptake of pravastatin is highly dependent on organic anion transporting polypeptide (human: OATP, rodent: Oatp) and subsequently excreted in unchanged form to the bile by multidrug resistance-associated protein 2 (human: MRP2, rodent: Mrp2). Hepatic transporters are key determinants for pharmacokinetics and pharmacodynamics and are therefore a likely target for possible PK drug-drug interactions.

The development of NASH is often surrounded by a pathological context with other comorbidities, such as cardiovascular diseases, obesity, insulin resistance, or type 2 diabetes mellitus. Given the complex pathophysiology and substantial heterogeneity of disease phenotypes, patients with NASH usually take multiple drugs including herbs. Evodiamine is a quinazoline alkaloid isolated from the fruit of *Evodia rutaecarpa* (Juss) Benth (Rutaceae) which is known as the traditional Chinese herb Wu-Zhu-Yu used for the treatment of gastrointestinal disorders, headache, and hypertension (Liao et al. 2011). Growing evidence demonstrates that evodiamine exerts anti-obesity (Bak et al. 2010), anti-inflammatory (Lv et al. 2015), analgesic (Wu and Chen 2019), anti-cardiovascular (Wei et al. 2013), and hypoglycaemic (Wang et al. 2013) activities. Due to the anti-inflammatory and cardiovascular protective effects of evodiamine, NASH patients who receive stains may have a simultaneous intake of evodiamine-containing herbs or natural products (Takahashi et al. 2015). Consequently, potential drug-drug or herb-drug interactions are likely to occur, which can lead to severe and sometimes lethal outcomes. However, there is a general lack of reliable information regarding such potential interactions. Therefore, it is important to highlight pharmacokinetic herb-drug interactions and to understand their mechanisms for the appropriate treatment of NASH patients with pravastatin.

In the present study, we investigated the possible interactions between pravastatin and evodiamine in MCD diet-induced NASH rats using *in vivo*, *in situ*, and *in vitro* experimental techniques. Moreover, we also evaluated the changes in the expression of Oatp and Mrp2 in the liver and explored the potential mechanisms with regard to these changes.

## Materials and methods

### Materials

Evodiamine (purity > 99.0%), pravastatin sodium (purity > 98.0%) and diclofenac (Internal standard, purity >98.0%) were purchased from Shanghai Aladdin Biochemical Technology Co., Ltd. (Shanghai, China). Oatp1a1 (PA5-97061) and Oatp1a4 (PA5-113548) primary antibodies were purchased from Thermo Fisher, USA. Oatp1b2 (sc-376904), Mrp2 (sc-59611), and β-actin (sc-8432) primary antibodies were provided by Santa Cruz Biotechnology (Shanghai, China). Alanine aminotransferase (ALT) ELISA kits, aspartate aminotransferase (AST) ELISA kits, and creatine kinase (CK) ELISA kits were purchased from Nanjing Jiancheng Biotech Co. (Nanjing, China). IL-1β ELISA kits, IL-6 ELISA kits, and TNF-α ELISA kits were purchased from Wuhan CUSABIO Biological Engineering Co., Ltd. (Wuhan, China). The HPLC grade acetonitrile and methanol were purchased from Thermo Fisher Scientific (China) Co., Ltd. (Shanghai, China).

### Animals and NASH model

Male Sprague-Dawley rats (8 weeks) were purchased from Beijing Vital River Laboratory Animal Technology Company Limited (Beijing, China). The rats were housed in a conditioned animal quarter at a temperature of 22–24 °C with a 12 h light/dark cycle and were allowed water and standard laboratory diet *ad libitum* during the 1-week acclimation period. All experimental procedures were conducted in conformity with the National Institute of Health guidelines regarding the principles of animal care. All animal experiments were approved by the Animal Ethics Committee of Henan Provincial Academy of Traditional Chinese Medicine (HNZYYYJ 20190116).

NASH was induced by feeding an MCD diet (l-amino acids 171.4 g/kg, sucrose 450.3 g/kg, corn starch 150.0 g/kg, dextral maltose 50.0 g/kg, cellulose 30.0 g/kg, corn oil 100.0 g/kg, sodium bicarbonate 7.5 g/kg, mineral mixture 35.0 g/kg, vitamin mixture 10.0 g/kg, Xietong Co., Ltd, Nanjing, China) to rats for 8 weeks, while the normal control rats were fed with the same diet (16% protein, 62% carbohydrate, and 21% fat) supplemented with l-methionine (3 g/kg) and choline (2 g/kg). The MCD diet is the best rat model for assessing alterations in drug transporters and drug disposition observed in clinical NASH (Canet et al. 2014). After the modelling was completed, two rats in the normal group and the model group were sacrificed. The liver histopathological scores of the two groups of rats were compared and comprehensively analysed to validate whether the model was successfully induced. Bodyweight and food intake were measured every week.

### Serum and liver biochemical parameters

The NASH rats were randomly divided into four groups with 6 rats: NASH group; NASH group treated with 10 mg/kg evodiamine; NASH group treated with 10 mg/kg pravastatin; NASH group treated with 10 mg/kg evodiamine and 10 mg/kg pravastatin. The normal rats were randomly divided into two groups with 6 rats: normal group; normal group treated with 10 mg/kg evodiamine. The dosages of evodiamine and pravastatin used in this study were cited from the reports (Crespo and Quidgley 2015; Eraslan et al. 2019). An equal volume of distilled water, evodiamine, or pravastatin was administered to the appropriate rats by oral gavage once daily for 14 days. Under isoflurane anaesthesia, blood samples from the abdominal aorta were collected 24 h after the last drug treatment and centrifuged at 1 500 *g* for 10 min at 4 °C. The levels of ALT and AST were determined as markers for hepatotoxicity following instructions for the procedure provided with the ELISA kits. The level of CK was detected as a marker for myotoxicity using ELISA kits. Liver samples were collected and washed with 0.9% saline, dried with filter paper, and weighed. A part of the liver tissue was homogenised in 0.9% saline using a homogeniser and centrifuged at 3 000 *g* for 10 min at 4 °C and resultant supernatant was used for measurement of IL-1β, IL-6, and TNF-α. The inflammatory cytokines were determined following instructions for the procedure provided with the ELISA kits.

### Histopathological analysis

A part of the fresh liver tissue was fixed in 4% paraformaldehyde for 24 h, embedded in paraffin, sectioned in 5 μm thick slides, and stained with haematoxylin and eosin (H&E). Histology was assessed blindly by a pathologist. The severity of the NASH of each group was evaluated according to the NASH activity scoring (NAS) system as previously described (Kleiner et al. 2005). The NAS scores were obtained from the unweighted sum of the histological components: steatosis (0–3), lobular inflammation (0–2), and hepatocellular ballooning (0–2). NASH was diagnosed when the NASH score was ≥4. Liver fibrosis was detected with Masson's trichrome staining, the percent of blue-stained area out of the total area of the liver section was calculated as the fibrosis index (%) using Image-J software.

### Pharmacokinetic experiments

For oral administration, the normal and NASH rats were divided into two groups, respectively: pravastatin alone (*n* = 6) and pravastatin co-administrated with evodiamine (*n* = 6), respectively. After pre-treatment with distilled water or 10 mg/kg evodiamine via gastric gavage for 14 days, pravastatin (10 mg/kg) was orally given to the rats after administration of distilled water or evodiamine on day 14. The dosages of evodiamine and pravastatin used in this study were cited from the reports (Crespo and Quidgley 2015; Eraslan et al. 2019). Blood samples (0.2 mL) were collected via the oculi choroidal vein at 0, 5, 15, 30, 45, 60, 90, 120, 240, 360 and 480 min after oral pravastatin.

For intravenous administration, another batch of rats was used. Evodiamine (5 mg/kg) or 0.9% saline was injected via the tail vein for 14 days. On day 14, the common bile duct of each rat was cannulated with a polyethylene tube (PE-10) under ether anaesthesia for bile sampling. Pravastatin (2 mg/kg) was immediately injected into the rats after injection of evodiamine. Blood samples (0.2 mL) were collected at 0, 5, 10, 20, 30, 45, 60, and 90 min post-pravastatin dosing. Then plasma was obtained by centrifugation at 3 000 *g* for 10 min. Bile samples were collected in tubes at a 15-min interval for 60 min and the bile volume of each time interval was measured. The dosages of pravastatin and evodiamine were cited from the reports (Li et al. 2015; Raffoul-Orozco et al. 2018) and reports (Tsai et al. 1995; Zhou et al. 2017), respectively. Samples of bile and plasma were stored at −80 °C until LC-MS/MS analysis.

### *In situ* single-pass intestinal perfusion

The effects of evodiamine on pravastatin absorption were evaluated by *in situ* single-pass perfusions. Briefly, the normal and NASH rats were pre-treated with distilled water or evodiamine (10 mg/kg) via gastric gavage for 14 days, then anaesthetised with isoflurane. The abdomen was opened with a midline incision. A small intestinal (duodenal, jejunum, or ileum) segment of approximately 10 cm or a colonic segment of approximately 5 cm was carefully isolated, then both ends were cannulated using rubber tubing and fixed by ligation. The isolated intestinal segments were softly washed with 0.9% saline (37 °C) to clear the contents. Then, the warm Krebs-Ringer solution free of pravastatin was pumped by a peristaltic pump at a flow rate of 0.2 mL/min for balancing 20 min, and then Krebs-Ringer buffer containing pravastatin (10 μg/mL) was replaced. After getting to a steady-state (10 min), the perfusate samples were continuously collected from the outlet of the duodenum, jejunum, ileum, and colon at 15 min intervals for 90 min. At the end of the experiments, the animals were euthanized and the areas of perfused intestinal segments were measured. All samples were stored at −80 °C until drug analysis.

The gravimetric method was employed to determine the net water flux. The effective permeability coefficient (P_eff_) was calculated using the mathematical expressions as follows: P_eff_ = −Qln (C_out_V_out_/C_in_V_in_)/2πRL, where C_in_ and C_out_ are the concentration of pravastatin in the perfusion solution through the inlet and outlet, V_in_ and V_out_ represent the inlet and outlet volume of perfusion solution, Q is the flow rate (0.2 mL/min) of perfusion solution, R is the radius of the intestinal segment, and L is the length of the intestinal segment.

### Tissue distribution studies

The normal and NASH rats intravenously received evodiamine (5 mg/kg) or 0.9% saline via the tail vein for 14 days. After evodiamine injection on day 14, pravastatin (2 mg/kg) was injected into the rats. The experimental rats were anaesthetised with isoflurane at 30 min after intravenous administration of pravastatin. Blood was collected from the abdominal aorta, then the liver, kidney, and soleus tissues were removed, weighed, and homogenised in 0.9% saline with the ratio of 1:4. Tissue homogenates were stored at −80 °C until further analysis.

### Uptake study using freshly isolated rat hepatocytes

The normal and NASH rats were administered distilled water or evodiamine (20 mg/kg) via gastric gavage for 14 days, then freshly primary hepatocytes of the experimental rats were isolated by Seglen's two-step collagenase IV perfusion method with slight modification as previously reported. Cell viability was greater than 85% through the Trypan blue exclusion test. Isolated hepatocytes were suspended in Hank's balanced salt solution (HBSS) at a density of 2.0 × 10^6^ cells/mL for the uptake study.

Following 5 min pre-incubation with HBSS at 37 °C, the uptake reaction was initiated by adding pravastatin (30 μM) to the cell suspension. Then cells were subjected to incubation at 37 °C for 2 and 5 min. The reaction was terminated by removing the drug-containing culture medium slowly. The cells were washed three times with ice-cold HBSS and lysed with 1% Triton X-100. Protein content was measured for each well using Pierce BCA Protein Assay Kit, and the accumulation of pravastatin in rat primary hepatocytes was analysed by UPLC-MS/MS analysis.

### Quantitative real-time polymerase chain reaction (qRT-PCR) analysis

The normal and NASH rats were divided into two groups, respectively: the control group (*n* = 6) and the evodiamine group (*n* = 6), respectively. After treatment with distilled water, or 10 mg/kg evodiamine via gastric gavage for 14 days, rats were sacrificed and livers were collected for quantitative real-time PCR and Western blotting analysis. Total RNA was extracted from the liver using Trizol reagent (Takara, Japan) according to the manufacturer's instructions and was reverse-transcribed into cDNA using the PrimeScript RT reagent kit (Takara, Japan). Quantitative real-time PCR analysis was performed using SYBR Green Master Mix (Thermo Fisher, Lithuania) and an ABI Prism 7000 Sequence Detector system. PCR amplification included a step at 95 °C for 30 s followed by 40 cycles of 15 s at 95 °C and 30 s at 60 °C. The following primers (Sangon Biotech, China) for target genes were used: Oatp1a1, forward 5′-ACCTGGAACAGCAGTATGGAAAA-3′ and reverse 5′-ACCGATAGGCAAAATGCTAGGTAT-3′; Oatp1a4, forward 5′-TGTGATGACCGTTGATAATTTTCCA-3′ and reverse 5′-TTCTCCACATATAGTTGGTGCTGAA-3′; Oatp1b2, forward 5′-CCTGTTCAAGTTCATAGAGCAGCA-3′ and reverse 5′-TGCCATAGTAGGTATGGTTATATTTC-3′; Mrp2, forward 5′-CAGGCCGGATTGTGAACAGA-3′ and reverse 5′-AGTGCCAGCGATGCCAAAG-3′; Gapdh, forward 5′-ATGGGAAGCTGGTCATCAAC-3′ and reverse 5′-GTGGTTCACACCCATCACAA-3′. The relative expressions of mRNA were analysed using the 2^−ΔΔCT^ method with Gapdh as the endogenous standard.

### Western blot analysis

The liver tissue was homogenised in cold lysis buffer (Solarbio Biotechnology, Beijing, China), and then centrifuged at 12 000 *g* for 10 min at 4 °C. The protein concentration was evaluated using BCA Protein Quantitative Kit (Solarbio Biotechnology, Beijing, China). Equal amounts of protein for each sample were separated by electrophoresis on sodium dodecyl sulfate-polyacrylamide gel electrophoresis (SDS-PAGE), followed by transfer to polyvinylidene fluoride (PVDF) membranes. Blocking of the membranes was performed for 2 h using 5% milk powder diluted in Tris-buffered saline with 0.1% Tween-20 (TBST). After three washes in TBST, membranes were incubated overnight at 4 °C with primary antibodies against Oatp1a1 (1:800), Oatp1a4 (1:800), Oatp1b2 (1:600), Mrp2 (1:600) and β-actin (1:1000). The membranes were washed three times with TBST and incubated with horseradish peroxidase-conjugated secondary antibodies (1:8000) at room temperature for 1 h. Blots were developed using enhanced chemiluminescence. Protein bands were captured with a FluorChem R Imaging System (ProteinSimple, USA) and analysed with Image-J software.

### LC-MS/MS analysis

The concentrations of pravastatin were determined using a Waters Acquity UPLC system coupled to Xevo TQS triple quadrupole mass spectrometer (Waters, Milford, USA). Following plasma, perfusate, bile, cell lysates, or tissue homogenate samples preparation, 500 μL of *n*-butanol saturated with water containing 50 μg/L diclofenac (internal standards) was mixed with 100 μL samples. After vortex mixing for 5 min and centrifuging at 12 000 *g* for 10 min, the organic layer was transferred and dried under a stream of nitrogen in an analytical evaporator (Thermo, Waltham, MA). The residue was dissolved in 100 μL of methanol and vortexed. Aliquots of the samples (5 μL) were injected into the liquid chromatography-mass spectrometry (LC-MS) system. Chromatographic separation was achieved on a Waters BEH C_18_ column (2.1 mm × 100 mm, 1.7 μm) with a mobile phase consisting of acetonitrile and water containing 0.1% formic acid (40:60, v/v) at a flow rate of 0.2 mL/min. The MS parameters were set as follows: ion spray voltage, −4500 V; source temperature, 500 °C; curtain gas, 20 L/h; and collision gas, 12 L/h. The mass spectrometric analysis was carried out on electrospray ionisation (ESI) source in negative ion mode, and the quantification was performed using multiple reaction monitoring (MRM) mode with m/z 423.4–320.9 for pravastatin and m/z 296.0–250.2 for diclofenac.

## Statistical analysis

The pharmacokinetic parameters including terminal plasma half-life (t_1/2_), apparent volume of distribution (V/F), clearance (CL/F), area under the plasma concentration-time (AUC), mean residence time (MRT), peak drug concentration (*C*_max_), and time to reach peak concentration (*T*_max_) were calculated using DAS software version 3.1 (Mathematical Pharmacology Professional Committee of China, Shanghai, China). Bioavailability was calculated according to the equation: bioavailability (%) = 100 × (AUC_oral_/Dose_oral_)/(AUC_intravenous_/Dose_intravenous_). Statistical analysis was performed using SPSS 22.0 (IBM Co, Armonk, USA). All results were expressed as mean ± SD and the differences between groups were analysed using one-way analysis of variance (ANOVA) followed by Student-Newman-Keuls multiple comparison *post hoc* test. Values of *p* < 0.05 were considered significant.

## Results

### Bodyweight, food intake, and liver weight

**Figure 1.**
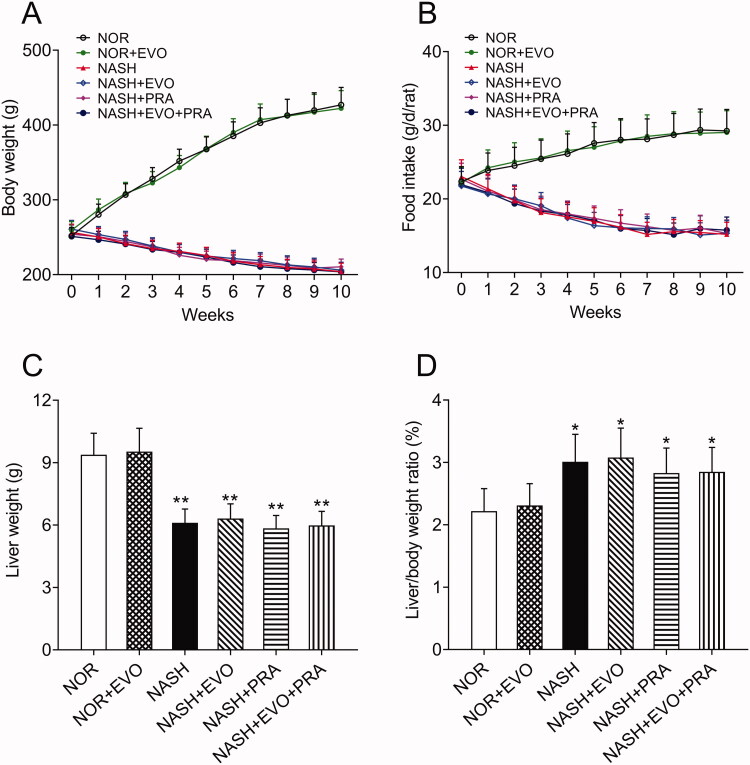
Effect of MCD diets on body weights (A), food intake (B), liver weights (C), and liver/body weight ratio (D) in rats. Data represent the mean ± SD of six rats. *p < 0.05, **p < 0.01 vs. Normal group. NOR, normal; EVO, evodiamine; PRA, pravastatin.

The body weights of the normal rats showed an increasing trend, while the body weights of rats decreased by approximately 20% following 8 weeks of MCD diet administration. In addition, the food intake of MCD diet-fed rats decreased gradually. The body weights and food intake of MCD diet-fed rats did not differ between any treated and untreated group during the next 2 weeks of treatment (Figure 1(A,B)). In all the MCD diet-fed rats, liver weights were significantly lower, whereas the liver/body weight ratios were higher than that of normal rats. We observed that any treatment did not significantly influence the liver weights and liver/body weight ratios of MCD diet-fed rats (Figure 1(C,D)).

### Effect of evodiamine on the biochemical parameters

**Figure 2.**
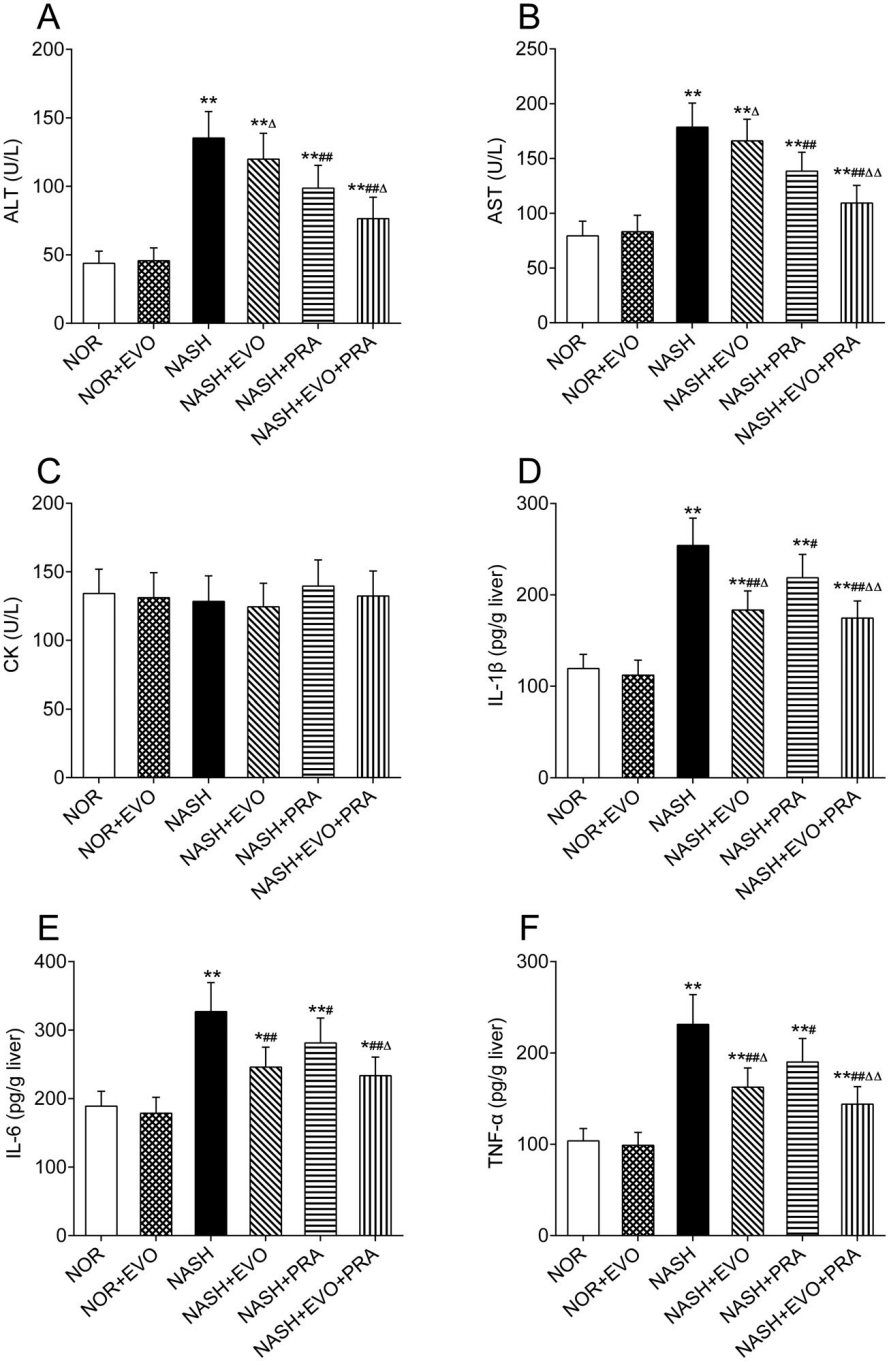
Effects of evodiamine alone and co-administered with pravastatin on the levels of ALT (A), AST (B), CK (C), IL-1β (D), IL-6 (E) and TNF-α (F). Data represent the mean ± SD of six rats. *p < 0.05, **p < 0.01 vs. Normal group; ^#^*p* < 0.05, ^##^*p* < 0.01 vs. NASH group; ^Δ^*p* < 0.05, ^ΔΔ^*p* < 0.01 vs. NASH + PRA group. NOR, normal; EVO, evodiamine; PRA, pravastatin.

The ALT, AST, CK, hepatic IL-1β, IL-6, and TNF-α levels in rats are shown in Figure 2. Compared with those in the normal group, the levels of ALT (135.27 ± 19.32 U/L), AST (178.64 ± 21.87 U/L), hepatic IL-1β (254.06 ± 29.82 pg/g liver), IL-6 (326.93 ± 42.55 pg/g liver), and TNF-α (231.24 ± 32.66 pg/g liver) increased significantly in the NASH rats. Pravastatin alone or co-administered with evodiamine reduced the elevated levels of ALT, AST, IL-1β, IL-6, and TNF-α. Furthermore, the levels of ALT, AST, hepatic IL-1β, IL-6, and TNF-α in the group treated with pravastatin plus evodiamine were lower than that of the rats treated with pravastatin alone. Pravastatin plus evodiamine was significantly superior to pravastatin alone in decreasing the levels of ALT (76.39 ± 15.70 vs. 98.71 ± 16.55 U/L), AST (109.29 ± 16.03 vs. 138.41 ± 17.28 U/L), IL-1β (174.53 ± 18.78 vs. 218.71 ± 25.41 pg/g liver), IL-6 (233.52 ± 26.98 vs. 281.26 ± 36.33 pg/g liver), and TNF-α (144.01 ± 19.17 vs. 190.03 ± 25.69 pg/g liver). We observed that evodiamine did not markedly influence the ALT, AST of NASH rats, but significantly reduced the contents of hepatic IL-1β, IL-6, and TNF-α by 27.82%, 24.76%, and 29.72%, respectively. However, there was no significant difference in CK among groups. These results indicated that pravastatin did not cause hepatotoxicity and myotoxicity either given alone or co-administration with evodiamine, and the combined administration attenuated hepatocellular inflammation more effectively than pravastatin alone in NASH rats.

### NASH histology and pathological assessment

**Figure 3.**
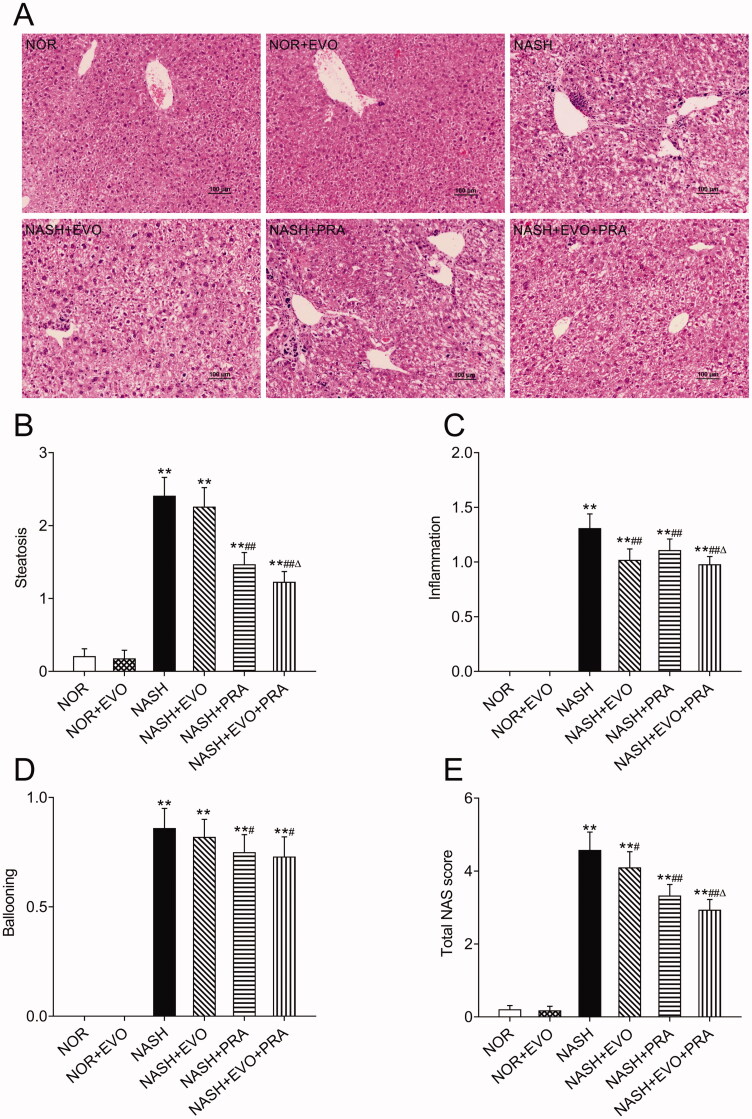
Liver histopathology and pathology scoring of NASH rats. (A) Representative haematoxylin and eosin-stained liver sections. (B) Steatosis score. (C) Inflammation score. (D) Ballooning score. (E) Total NAS score. Data represent the mean ± SD of six rats. *p < 0.05, **p < 0.01 vs. Normal group; ^#^*p* < 0.05, ^##^*p* < 0.01 vs. NASH group; ^Δ^*p* < 0.05, ^ΔΔ^*p* < 0.01 vs. NASH + PRA group. NOR, normal; EVO, evodiamine; PRA, pravastatin.

H&E stained liver sections from rats are shown in Figure 3. The liver sections from MCD diet-fed rats showed extensive macrovesicular fat accumulation and inflammatory infiltration, which are common pathological lesions in NASH. H&E stained sections were evaluated according to a previously validated NASH pathology scoring rubric. NASH was defined as a NAS score greater than or equal to 4. This evaluation demonstrated that MCD diet-fed rats have NASH. The NASH activity score decreased significantly after pravastatin alone or co-administered with evodiamine administration. Furthermore, the NASH activity scores in the rats treated with pravastatin plus evodiamine were lower than that of the rats treated with pravastatin alone.

**Figure 4.**
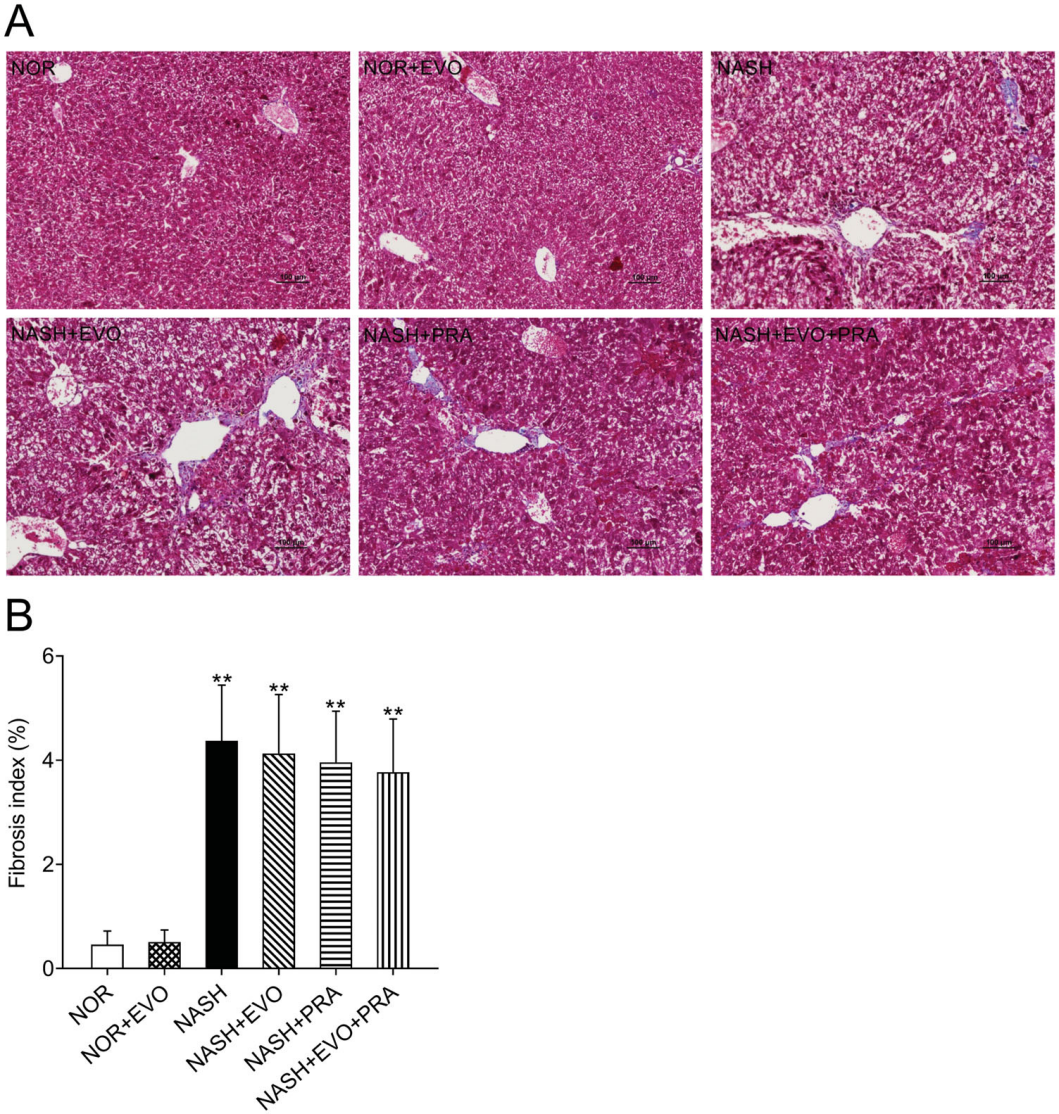
Assessment of liver fibrosis in the different experimental groups by Masson's trichrome staining. (A) Representative Masson's trichrome stained liver sections. (B) Fibrosis index. Data represent the mean ± SD of six rats. *p < 0.05, **p < 0.01 vs. Normal group; ^#^*p* < 0.05, ^##^*p* < 0.01 vs. NASH group; ^Δ^*p* < 0.05, ^ΔΔ^*p* < 0.01 vs. NASH + PRA group. NOR, normal; EVO, evodiamine; PRA, pravastatin.

The extent of liver fibrosis was evaluated using Masson's trichrome staining, which stains collagen fibres blue. The results clearly showed that MCD diet-fed rats displayed signs of fibrosis including periportal and interstitial collagen deposition. Quantification suggested that the MCD diet-induced a fibrosis index of 4.37%, and any treatment did not significantly change the fibrotic severity (Figure 4).

### Effect of evodiamine on the pharmacokinetics of pravastatin

**Figure 5.**
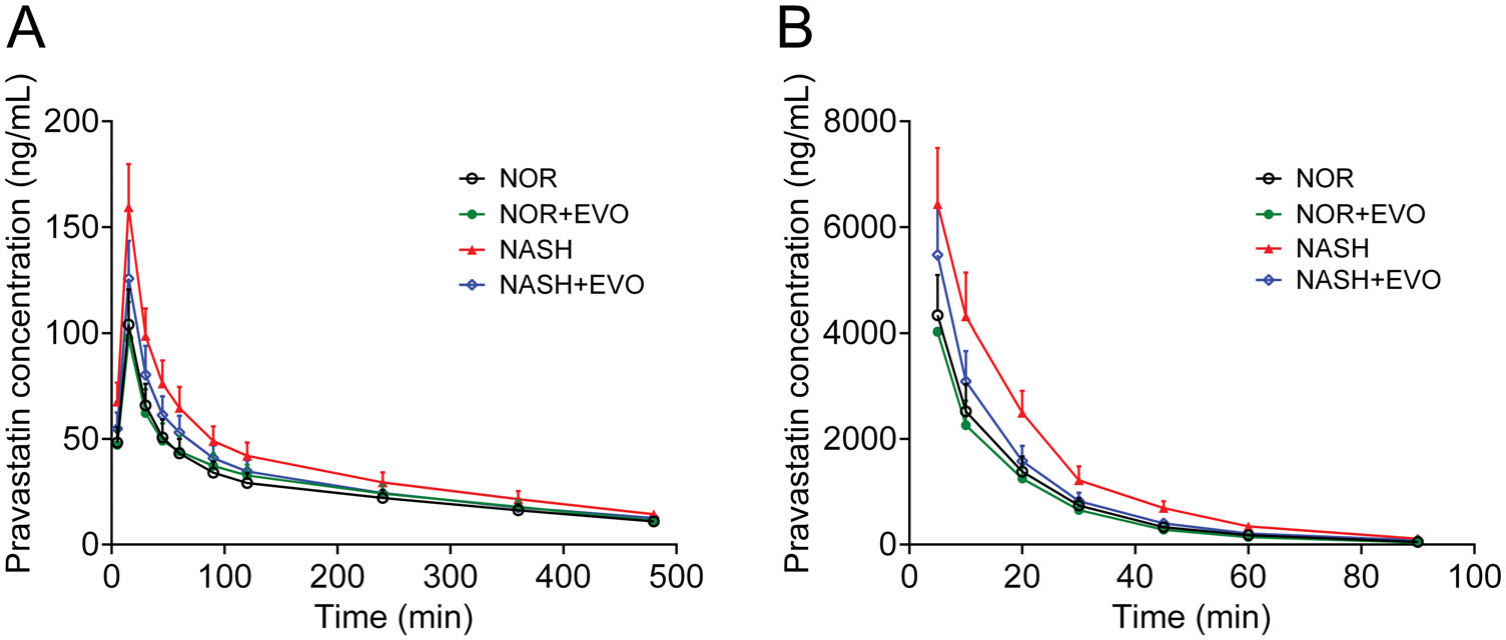
The plasma concentration-time curves of pravastatin in normal and NASH rats. (A) Rats have received pravastatin (10 mg/kg) orally alone or with the pre-treatment of evodiamine (10 mg/kg). (B) Rats were administered pravastatin (2 mg/kg) intravenously alone or with the pre-treatment of evodiamine (5 mg/kg). Each point represents the mean ± SD of six rats. NOR, normal; EVO, evodiamine.

The mean blood concentration-time curves of pravastatin in normal and NASH rats after oral or intravenous administration of pravastatin with or without the pre-treatment of evodiamine are shown in Figure 5, and the corresponding pharmacokinetic parameters are described in Table 1. The results showed that evodiamine exhibited different effects on the systemic exposures of pravastatin in normal and NASH rats.

**Table 1.** Pharmacokinetic parameters of pravastatin with or without pre-treatment of evodiamine in normal and NASH rats.

Parameters	NOR	NOR + EVO	NASH	NASH + EVO
Oral				
AUC_0-t_ (mg/min/L)	12.73 ± 1.68	13.52 ± 1.81	18.17 ± 2.52**	14.91 ± 2.03^#^
AUC_0-∞_ (mg/min/L)	16.51 ± 2.06	18.70 ± 2.20	22.99 ± 2.62**	19.50 ± 2.31^#^
MRT_0-t_ (min)	168.80 ± 20.06	172.19 ± 24.91	160.43 ± 22.09	162.26 ± 23.75
*t*_1/2_ (min)	238.45 ± 32.96	273.43 ± 40.18	232.18 ± 34.57	252.59 ± 37.10
*T*_max_ (min)	20.00 ± 7.75	17.50 ± 6.12	17.50 ± 6.12	22.5 ± 8.22
Vz/F (L/kg)	197.43 ± 25.65	208.97 ± 28.28	172.48 ± 21.07	187.76 ± 23.81
CL/F (mL/min/kg)	625.16 ± 112.38	587.60 ± 107.07	413.95 ± 92.73*	541.71 ± 101.84^#^
*C*_max_ (μg/L)	103.89 ± 18.36	97.62 ± 16.48	159.43 ± 26.63**	125.61 ± 22.17^#^
Intravenous				
AUC_0-t_ (mg/min/L)	84.75 ± 16.36	76.04 ± 15.09	138.09 ± 22.91**	107.62 ± 19.73^#^
AUC_0-∞_ (mg/min/L)	85.33 ± 16.91	76.82 ± 15.32	139.57 ± 23.24**	108.79 ± 19.95^#^
MRT_0-t_ (min)	15.14 ± 3.59	14.61 ± 3.78	16.88 ± 4.06	15.36 ± 3.81
*t*_1/2_ (min)	13.17 ± 3.13	15.19 ± 2.87	14.28 ± 2.79	15.26 ± 3.02
Vz (L/kg)	0.45 ± 0.08	0.49 ± 0.10	0.33 ± 0.07	0.40 ± 0.08
CL (mL/min/kg)	22.64 ± 4.77	25.07 ± 5.11	14.21 ± 3.26**	19.48 ± 3.82^#^

Data values are depicted as mean ± SD of six rats. *p < 0.05, **p < 0.01 vs. Normal group; ^#^*p* < 0.05, ^##^*p* < 0.01 vs. NASH group. NOR, normal; EVO, evodiamine.

In normal rats, there was no significant difference in any pharmacokinetic parameters of pravastatin between the pravastatin alone group and the co-administration group, which indicated that neither oral nor intravenous administration of evodiamine influenced the pharmacokinetics of pravastatin. In NASH rats, the AUC_0-t_, AUC_0-∞_, and *C*_max_ values of pravastatin after oral administration were 1.43-, 1.39-, and 1.53-fold of those in normal rats, respectively. The clearance in NASH rats was 66.22% of that in normal rats. For intravenous administration, the AUC_0-t_ and AUC_0-∞_ values of pravastatin in NASH were 1.63-, and 1.64-fold of those in normal rats, respectively. The clearance in NASH rats was 62.76% of that in normal rats. These results indicated the higher systemic exposure and lower clearance of pravastatin in NASH rats. Co-administration of evodiamine markedly decreased plasma concentrations of pravastatin, leading to significantly decreased systemic exposure. For oral administration, the AUC_0-t_, AUC_0-∞_, and *C*_max_ values of pravastatin in NASH rats with evodiamine were 82.06%, 84.81%, and 78.79% of those without evodiamine, respectively. For intravenous administration, co-administration of evodiamine significantly decreased the AUC_0-t_, and AUC_0-∞_ of pravastatin by 22.07% and 22.05%. Additionally, the clearance of pravastatin was enhanced significantly after evodiamine treatment in NASH rats. Additionally, neither NASH nor evodiamine significantly altered the bioavailability of pravastatin.

### Effect of evodiamine on the biliary excretion of pravastatin

**Figure 6.**
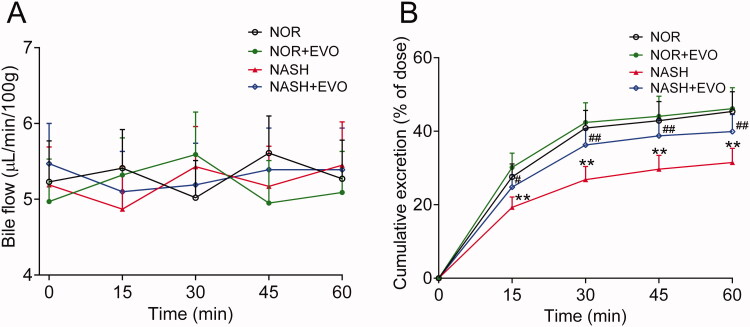
The effect of evodiamine on the biliary excretion of pravastatin. (A) Bile flow. (B) Cumulative biliary excretion of pravastatin. Data are presented as mean ± SD, *n* = 6. *p < 0.05, **p < 0.01 vs. Normal group; ^#^*p* < 0.05, ^##^*p* < 0.01 vs. NASH group. NOR, normal; EVO, evodiamine.

The effect of evodiamine on the biliary excretion of pravastatin was investigated following the intravenous dose (Figure 6). There were no significant differences in bile flow among groups at any of the time points. The cumulative biliary excretion of pravastatin in NASH rats was significantly lower than that in normal rats. Evodiamine markedly increased the biliary excretion of pravastatin in NASH rats but had no effect in normal rats.

### Effect of evodiamine on the absorption of pravastatin

**Figure 7.**
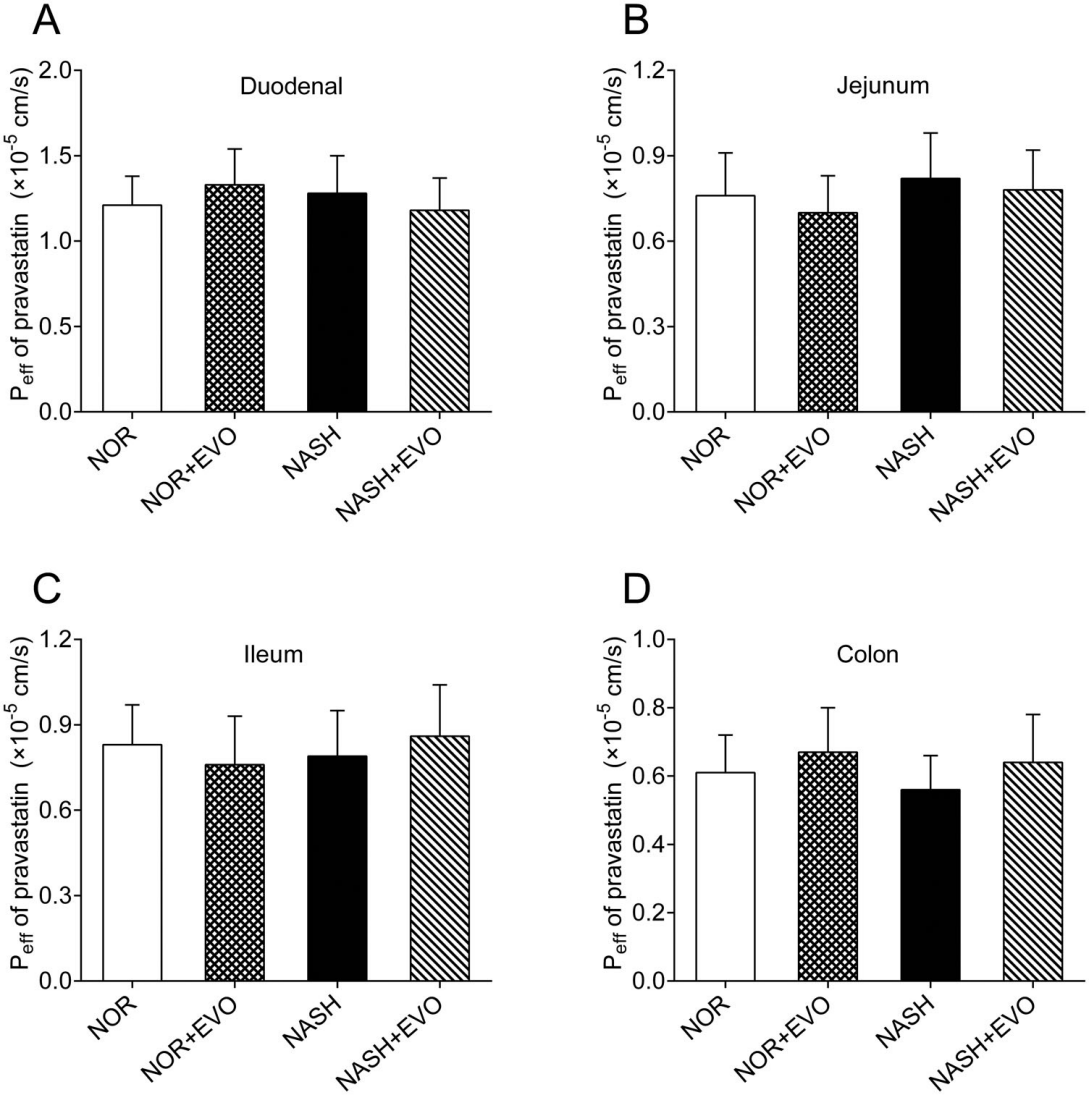
The effect of evodiamine on the absorption of pravastatin in the duodenum (A), jejunum (B), ileum (C) and, colon (D). Data represent the mean ± SD of six rats. NOR, normal; EVO, evodiamine.

The intestinal effective permeability coefficients of pravastatin with and without pre-treatment of evodiamine were measured using *in situ* single-pass intestinal perfusions (Figure 7). The results showed no significant difference in the Peff of pravastatin was obtained among the groups in duodenum, jejunum, ileum, and colon, indicating that evodiamine did not affect the intestinal absorption of pravastatin.

### Effects of evodiamine on the tissue distribution of pravastatin

**Figure 8.**
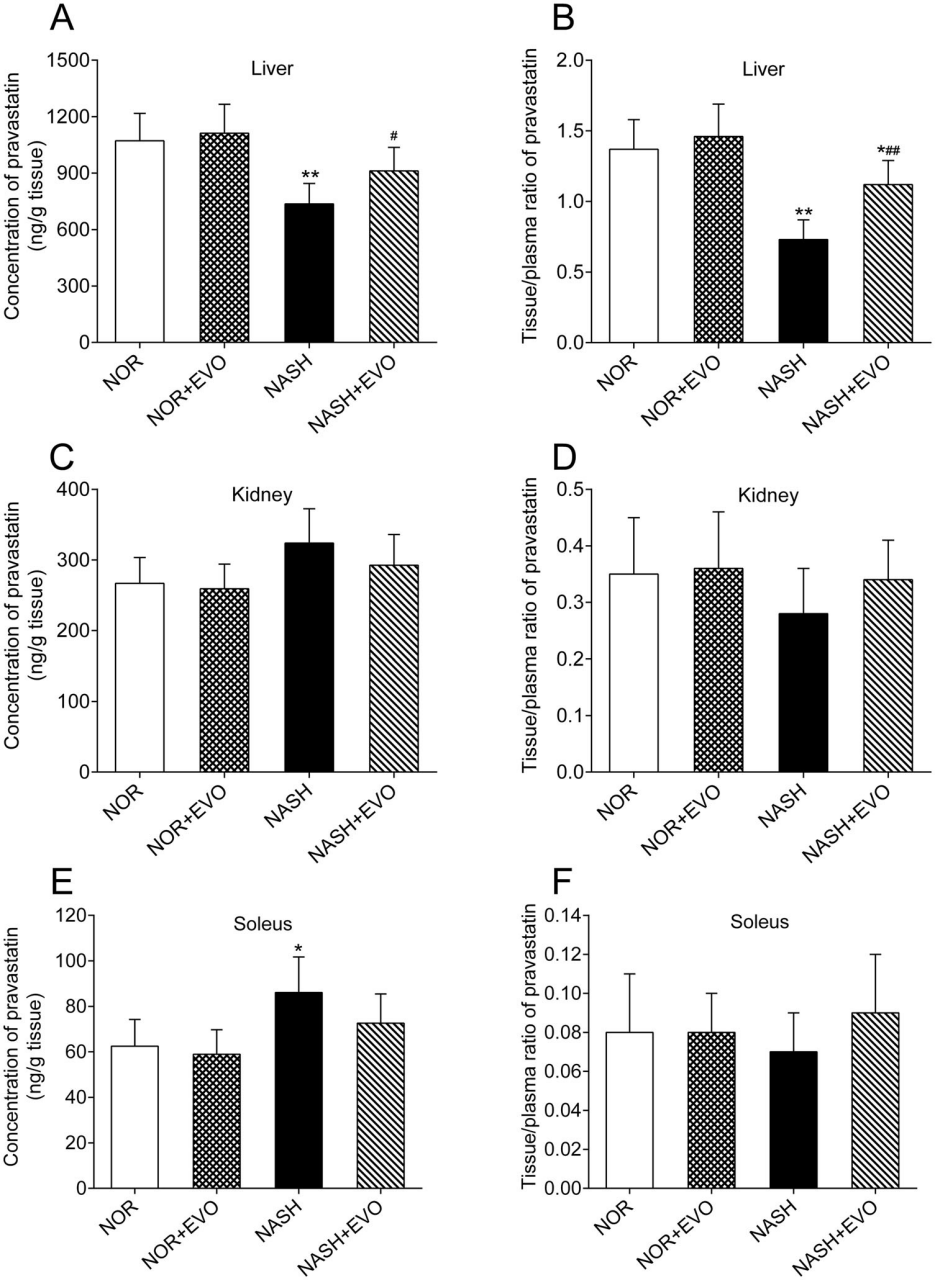
The effect of evodiamine on the tissue distribution of pravastatin in the liver (A,B), kidney (C,D) and soleus (E,F). Data represent the mean ± SD, *n* = 6. *p < 0.05, **p < 0.01 vs. Normal group; ^#^*p* < 0.05, ^##^*p* < 0.01 vs. NASH group. NOR, normal; EVO, evodiamine.

Pravastatin concentrations and the tissue to plasma (T/P) ratios in liver, kidney and soleus of normal, and NASH rats are presented in Figure 8. Results showed that the hepatic distribution and tissue to plasma ratio of pravastatin in NASH rats were 72.26% and 53.28% of those in normal rats, respectively. Pravastatin concentration in the soleus of NASH rats was 1.38-fold of that in normal rats. Effects of evodiamine on pravastatin distribution in the liver, kidney, and soleus of normal and NASH rats were also investigated. It was found that evodiamine could enhance the hepatic distribution and tissue to plasma ratio of pravastatin in NASH rats. Renal distribution and tissue to plasma ratio of pravastatin glucose did not differ among the groups. These data indicated that NASH reduced the hepatic distribution of pravastatin, and evodiamine treatment reversed this effect.

### Effect of evodiamine on hepatic uptake of pravastatin

**Figure 9.**
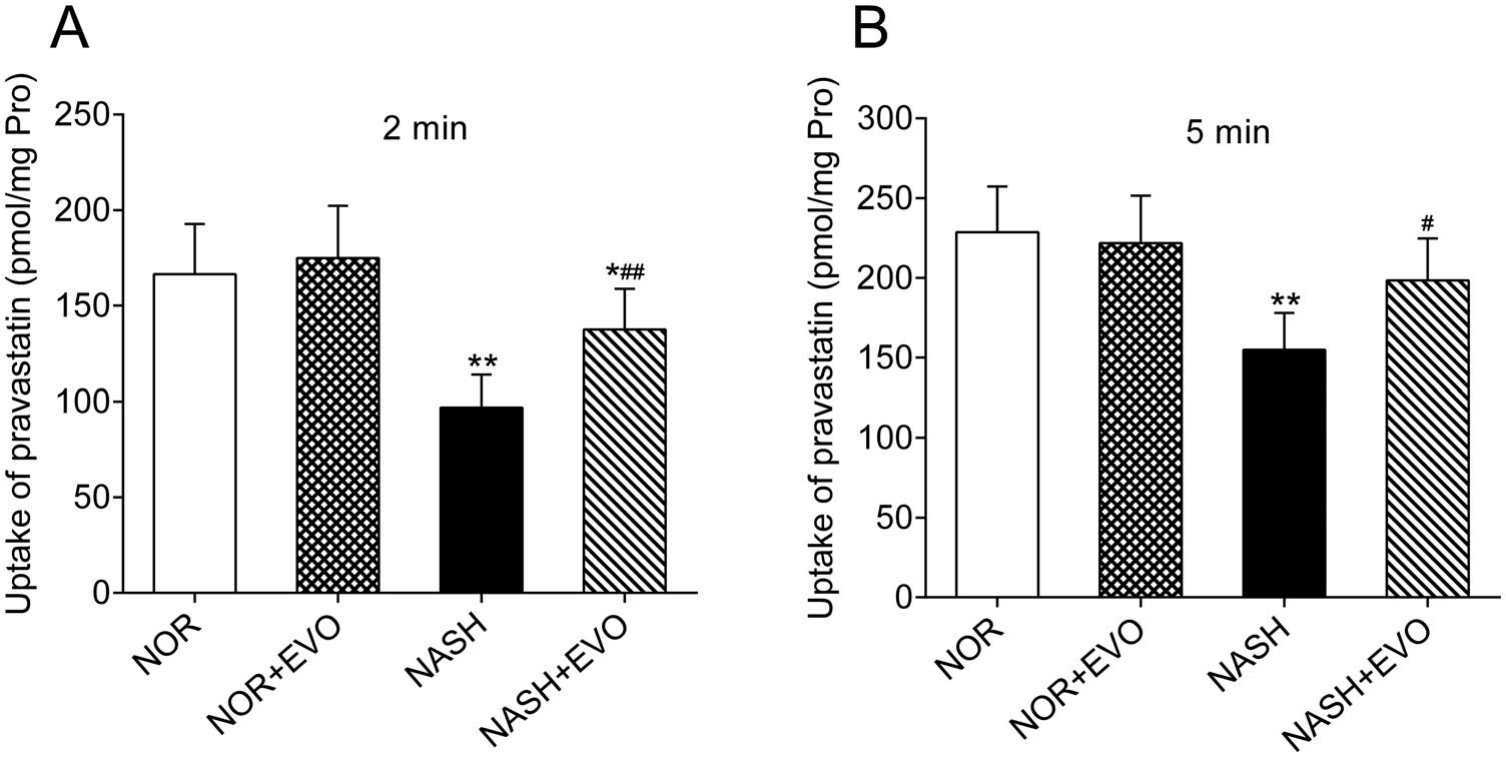
The effect of evodiamine on the uptake of pravastatin in primary rat hepatocytes. The incubation time was 2 (A) and 5 (B) min. Data represent the mean ± SD, *n* = 6. *p < 0.05, **p < 0.01 vs. Normal group; ^#^*p* < 0.05, ^##^*p* < 0.01 vs. NASH group. NOR, normal; EVO, evodiamine.

The effect of evodiamine on the uptake of pravastatin was investigated using freshly isolated primary hepatocytes of normal and NASH rats. As illustrated in Figure 9, compared to the hepatocytes from normal rats, the uptake of pravastatin significantly decreased in the hepatocytes from the NASH rats. The hepatocytes from NASH rats pre-treated with evodiamine exhibited higher uptake of pravastatin than the NASH rats. However, the uptake of pravastatin did not appear to differ between the normal rats and the normal rats pre-treated with evodiamine. These data indicated that evodiamine can enhance the uptake of pravastatin in rat primary hepatocytes from the NASH rats.

### Effects of evodiamine on mRNA levels of Oatp1a1, Oatp1a4, Oatp1b2, and Mrp2

**Figure 10.**
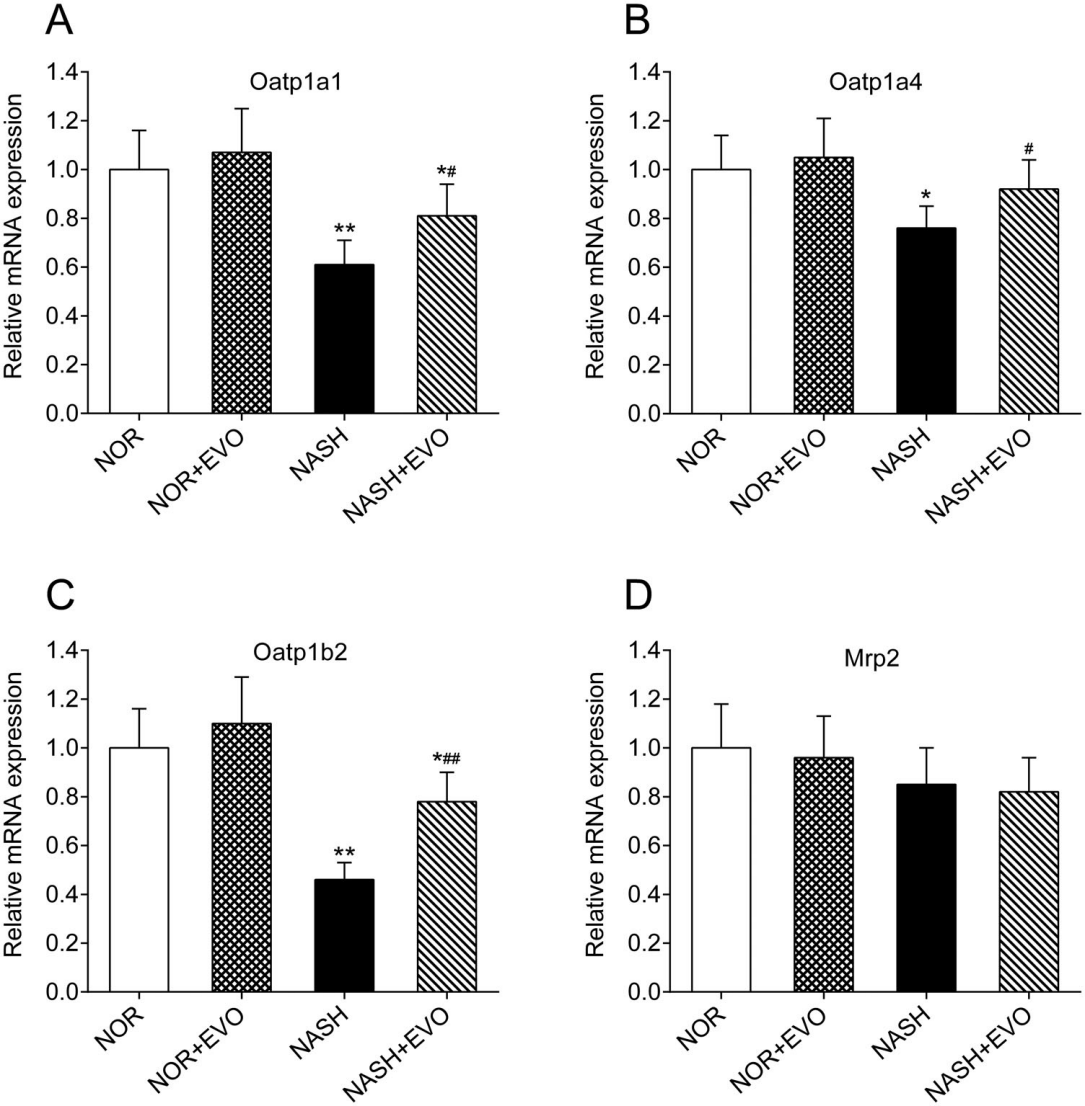
The effect of evodiamine on the mRNA expression of hepatic Oatp1a1 (A), Oatp1a4 (B), Oatp1b2 (C) and Mrp2 (D) in rats. Data represent the mean ± SD of six rats. *p < 0.05, **p < 0.01 vs. Normal group; ^#^*p* < 0.05, ^##^*p* < 0.01 vs. NASH group. NOR, normal; EVO, evodiamine.

The mRNA levels of Oatp1a1, Oatp1a4, Oatp1b2, and Mrp2 in the liver of normal and NASH rats were measured by quantitative real-time PCR. As shown in Figure 10, compared with the normal rats, significant decreases in hepatic Oatp1a1, Oatp1a4, and Oatp1b2 mRNA occurred in the liver of NASH rats. Administration of evodiamine significantly increased the mRNA expressions of Oatp1a1, Oatp1a4, and Oatp1b2. Moreover, mRNA levels of Mrp2 in the liver were comparable among all groups of rats. These results suggested that treatment with evodiamine significantly increased the hepatic uptake of Oatp1a1, Oatp1a4, and Oatp1b2 substrates in NASH rats.

### Effect of evodiamine on protein expressions of Oatp1a1, Oatp1a4, Oatp1b2, and Mrp2

**Figure 11.**
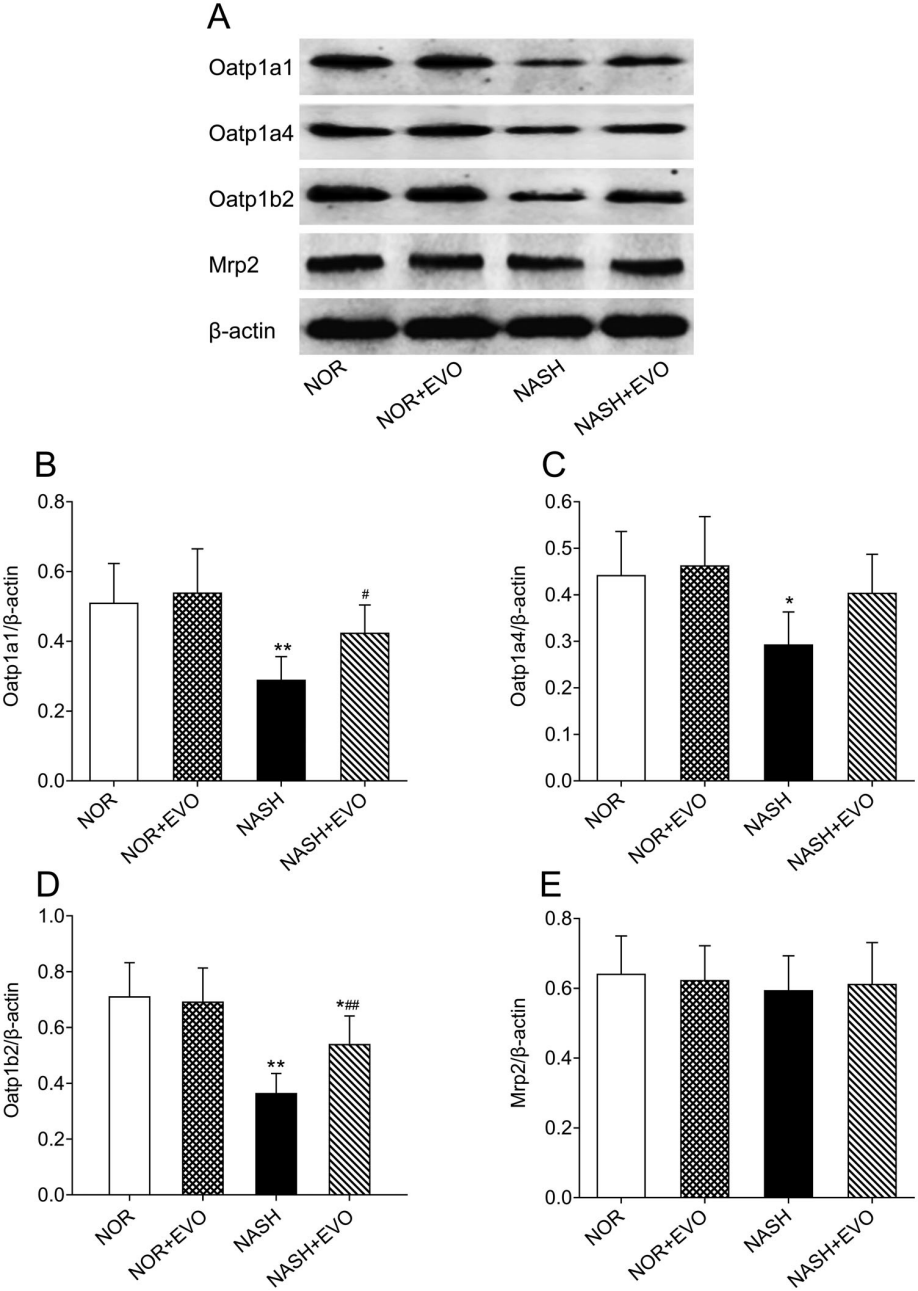
The effect of evodiamine on the protein expression of hepatic Oatp1a1, Oatp1a4, Oatp1b2 and Mrp2. (A) Representative photos of western blotting. (B) The relative intensity value of Oatp1a1. (C) The relative intensity value of Oatp1a4. (D) The relative intensity value of Oatp1b2. (E) The relative intensity value of Mrp2. Data represent the mean ± SD of six rats. *p < 0.05, **p < 0.01 vs. Normal group; ^#^*p* < 0.05, ^##^*p* < 0.01 vs. NASH group. NOR, normal; EVO, evodiamine.

The effect of evodiamine treatment on the expressional modulation of transporters in the liver responsible for pravastatin distribution and elimination was investigated by a western bolt. As shown in Figure 11, the protein expression of hepatic Oatp1a1, Oatp1a4, and Oatp1b2 decreased significantly in NASH rats compared with the normal group. Interestingly, evodiamine did not appear to have significant effects on the protein level of any transporter in normal rats but up-regulated protein expressions of Oatp1a1, Oatp1a4, and Oatp1b2 in NASH rats. The level of Mrp2 which is involved in the excretion of pravastatin into the bile was not altered in all groups. Western blot analysis of hepatic transporters suggested that the protein expression of uptake transporter decreased significantly and evodiamine could effectively reverse the down-regulation of Oatp1a1, Oatp1a4, and Oatp1b2.

## Discussion

In the present study, we investigated the effect of evodiamine on the pharmacokinetics of pravastatin and the related mechanisms. Firstly, we found evodiamine reduced the AUC and *C*_max_ values of pravastatin in NASH rats but had no significant effect on any pharmacokinetic parameter of pravastatin in normal rats. Secondly, we confirmed that the variety in the systemic exposure of pravastatin might be because evodiamine enhanced the hepatic distribution of pravastatin in NASH rats. Further, evodiamine increased the hepatic uptake of pravastatin was probably attributed to the upregulated Oatp in the liver. Finally, differential regulation of inflammatory factors was likely to be the underlying mechanism for the different effects of evodiamine on pravastatin uptake in normal and NASH rats.

Successful establishment of the NASH models is critical for this study. MCD diet is widely used in NASH animal studies and can induce the histological features of steatohepatitis in human NASH. Although the weight loss, low levels of blood lipids, and normal insulin sensitivity in the model are not consistent with the clinical metabolic characteristics of NASH, this model is generally considered adequate to study the intrahepatic events including transporters alteration (Clarke et al. 2014). In the current study, rats fed the MCD diet for 8 weeks developed hepatic steatosis, inflammation, and fibrosis, which suggested that the NASH model was successfully established.

The plasma pharmacokinetic study showed that administration of evodiamine reduced the AUC and *C*_max_ values of pravastatin in NASH rats, but had no significant effect on any pharmacokinetic parameter of pravastatin in normal rats. It is well known that the pharmacokinetics of a drug is determined by its absorption, distribution, metabolism, and excretion, alteration in any process may cause changes in systemic explore. The absorption rate of pravastatin from the intestine was reported to be relatively high (approximately 30%) (Mukhtar et al. 2005), the interaction between pravastatin and evodiamine might occur in the absorption stage. As physiological differences, including enzyme activities, amount and capacity of carriers, the thickness of mucous layers, membrane components, and so on, exist in different intestinal segments (Dahan et al. 2009), the extent of absorption might alter differently in different intestinal segments. So, we investigated the effect of evodiamine on the absorptive behaviour of pravastatin in the duodenum, jejunum, ileum, and colon by *in situ* single-pass intestinal perfusion. The data showed that the effective permeability coefficient of pravastatin in four different intestinal segments had no significant difference in all rats. To investigate whether the higher exposure of pravastatin came from decreased systemic clearance in NASH rats, the pharmacokinetics of pravastatin was also studied after intravenous administration. Following intravenous dose, the systemic clearance of pravastatin in NASH rats was decreased, the extent of decrease in systemic clearance was close to that following oral administration, inferring that the increased exposure of pravastatin after oral administration in NASH rats mainly came from an increase in systemic clearance. Additionally, the extent of decrease of systemic exposure by evodiamine following oral administration was almost the same as that following intravenous administration. Neither NASH nor evodiamine significantly altered the bioavailability of pravastatin. These results above indicated that the enhanced systemic exposure of pravastatin in NASH rats might be associated with its elimination rather than absorption, and evodiamine did not affect the absorption of pravastatin.

It is well accepted that systemic clearance of drugs generally involves three elimination routes: hepatobiliary excretion, renal excretion, and hepatic metabolism. Pravastatin has been demonstrated to undergo little metabolism by drug-metabolizing enzymes and is mainly excreted into bile in unchanged form (Hatanaka 2000; Watanabe et al. 2009). In addition, the excreted amount of pravastatin via intestinal fluid and urine was minor, less than 2% of the intravenous dose (Li et al. 2015). The current pharmacokinetic evaluation showed that evodiamine treatment could reverse the reduced systemic clearance of pravastatin in NASH rats, but had no influence in normal rats. We speculated that the differential effect of evodiamine on the systemic clearance and plasma exposure of pravastatin in normal and NASH rats might be attributable to the differential alteration in the hepatobiliary excretion of pravastatin by evodiamine. Effects of evodiamine on the hepatic uptake and bile excretion of pravastatin were investigated both *in vitro* and *in vivo*. The data of the *in vitro* experiment revealed that the uptake of pravastatin by the freshly isolated hepatocytes of NASH rats was consistently less than hepatocytes of normal rats. Evodiamine significantly enhanced hepatic uptake of pravastatin in the freshly isolated hepatocytes of NASH rats but had no effect in the hepatocytes of normal rats. The *in vivo* results showed that the cumulative biliary excretion of pravastatin in NASH rats was lower than that in normal rats, and evodiamine markedly increased the biliary excretion of pravastatin in NASH rats. The tissue distribution results showed that hepatic distribution and the liver to plasma ratios were significantly lowered in NASH rats compared to the normal rats, while evodiamine pre-administration significantly increased pravastatin concentration in the liver of NASH rats. However, evodiamine exerted no influence on pravastatin distributions and the tissue to plasma ratios in the liver of normal rats. These results above suggested that altered hepatobiliary excretion could be the cause of changed pharmacokinetics of pravastatin in NASH rats, evodiamine decreased the systemic exposure of pravastatin via enhancement its hepatobiliary excretion.

For drugs that are mainly eliminated by the liver, their plasma levels are mainly dependent on hepatic intrinsic clearance following oral administrations. The hepatic intrinsic clearance is affected by hepatic uptake (by transporters) and metabolism (by metabolic enzymes). The changes in the expression/activities of transporters and metabolic enzymes have significant impacts on the hepatic clearance of drugs. Previous studies have demonstrated that the OATP family (OATP1B1/1B3 in humans; Oatp la1, la4, and lb2 in rats) locating at the hepatocyte basolateral membrane mediates the hepatic uptake of pravastatin from blood, which directly affect the pravastatin systemic explore and liver selectivity (Kunze et al. 2014; Takano et al. 2018); MRP2/Mrp2 as an efflux transporter expressing at the canalicular membrane mainly mediates the biliary excretion of pravastatin and may account for the pravastatin level in blood indirectly (Ellis et al. 2013). In the present study, we found there was a significant difference in clearance of pravastatin between NASH and normal rats, which could be ascribed to the down-regulated expression of expressions of Oatp1a1, Oatpla4, and Oatplb2 rather than the unchanged expression of Mrp2 in the liver. The decreased systemic clearance of pravastatin further verified that the down-regulated Oatp-mediated hepatobiliary excretion reduction caused the elevation of systemic exposure for pravastatin in NASH rats. These findings were consistent with the previous study (Clarke et al. 2014), which suggested that the downregulation of the OATP family is a consequence of non-alcoholic steatohepatitis and OATPs mediating the hepatic uptake process is the crucial step in pravastatin pharmacokinetics and disposition. Interestingly, evodiamine exhibited different roles in the expression of OATPs in normal and NASH rats. In normal rats, neither Oatp mRNA levels nor protein expressions were affected by evodiamine, but in NASH rats, both mRNA and protein expression was significantly up-regulated by evodiamine. In this context, the up-regulation of Oatp1a1, Oatpla4, and Oatplb2 triggered the pravastatin concentration in the blood to decrease in NASH rats. All these results gave a clue that the hepatic disease state was likely to be a crucial player in the different regulation of hepatic uptake transporter expression by evodiamine.

The underlying mechanism for the down-regulation of Oatp expression in the liver of NASH rats has been described in detail (Fisher et al. 2009; Tanaka et al. 2012). The genes encoding for OATPs in the liver are regulated by a complex interacting network of hepatocyte nuclear factors (HNF1, 3, 4) and nuclear (orphan) receptors (e.g., farnesoid X receptor/bile acid receptor, pregnane X receptor, constitutive androstane receptor). Pro-inflammatory cytokines, drugs, and bile acids mediate causative and adaptive transporter changes at the transcriptional level by interacting with these nuclear factors and receptors (Geier et al. 2007). Treatment by the pro-inflammatory factor lipopolysaccharide (LPS) has been shown to result in a pronounced down-regulation of hepatic transporter expression in rodents (Cherrington et al. 2004; Yano et al. 2010). In human hepatocytes, exposure to TNF-α, IL-1β, or IL-6 was found to down-regulate mRNA levels of major sinusoidal influx transporters, including sodium taurocholate co-transporting polypeptide (NTCP), OATP1B1, OATP1B3, OATP2B1, organic cation transporter 1 (OCT1), and organic anion transporter 2 (OAT2). TNF-α and IL-6 concomitantly reduced NTCP and OATP1B1 protein expression and NTCP, OATP1B1, and OCT1 transport activities (Le Vee et al. 2008, 2009; Vee et al. 2011). Hepatic inflammation is one of the most distinguished features of NASH, the elevated pro-inflammatory cytokines levels have been shown to implicate in the pathogenesis and progression of NASH. The present experimental results demonstrated that MCD-induced rats showed increased levels of hepatocellular IL-1β, IL-6, and TNF-α, which might suggest a potential mechanism for the down-regulation of the Oatplal, Oatp1a4, and Oatp1b2 expressions in these animals accompanied by the NASH. It was also found that administration of evodiamine significantly decreased the levels of hepatic IL-1β, IL-6, and TNF-α in NASH rats, but did not significantly affect any of the three inflammatory factors in normal rats. All these results may explain our findings that evodiamine differentially regulated the expression of Oatp1a1, Oatp1a4, and Oatp1b2 in normal and NASH rats, and subsequently enhanced the hepatic uptake of pravastatin only in NASH rats.

Reports stated that statin-induced myotoxicity was better associated with its plasma concentrations (Mammen and Amato 2010). The plasma levels of pravastatin were statistically different among groups, but the value of CK (as a biomarker of myotoxicity) exhibited no prominent difference. This might be because the concentrations of pravastatin were not sufficiently high to cause myotoxicity in all experimental rats. Although evodiamine appreciably increased the hepatic distribution of pravastatin in NASH rats, serum ALT and AST levels, sensitive indicators of liver injury, decreased instead after co-administration of evodiamine and pravastatin, which was likely due to more remarkable therapeutic effects against NASH of combination administration.

## Conclusions

NASH can reduce the expression of various Oarps, which increases the plasma level of pravastatin. Co-administration of evodiamine increased the systemic exposure of pravastatin in NASH rats via enhancing hepatic uptake of pravastatin. Evodiamine increased the hepatic distribution of pravastatin due to the up-regulation of hepatic OATPs expressions, which might be associated with the decrease in inflammatory cytokines. Although the function and structure of transport proteins may vary between rats and humans, this study indicated that the efficacy of pravastatin may be altered in NASH patients when combined with evodiamine.
